# Global gene expression of *Poncirus trifoliata*, *Citrus sunki *and their hybrids under infection of *Phytophthora parasitica*

**DOI:** 10.1186/1471-2164-12-39

**Published:** 2011-01-17

**Authors:** Leonardo P Boava, Mariângela Cristofani-Yaly, Valéria S Mafra, Karen Kubo, Luciano T Kishi, Marco A Takita, Marcelo Ribeiro-Alves, Marcos A Machado

**Affiliations:** 1 Centro APTA Citros Sylvio Moreira, CP4, 13490-970, Cordeirópolis-SP, Brazil; 2 Centro de Desenvolvimento Tecnológico em Saúde, Fundação Oswaldo Cruz. Av. Brasil, 4365 - 21040-900 - Rio de Janeiro-RJ, Brasil

## Abstract

**Background:**

Gummosis and root rot caused by *Phytophthora *are among the most economically important diseases in citrus. Four F_1 _resistant hybrids (Pool R), and four F_1 _susceptible hybrids (Pool S) to *P. parasitica*, were selected from a cross between susceptible *Citrus sunki *and resistant *Poncirus trifoliata *cv. Rubidoux. We investigated gene expression in pools of four resistant and four susceptible hybrids in comparison with their parents 48 hours after *P. parasitica *inoculation. We proposed that genes differentially expressed between resistant and susceptible parents and between their resistant and susceptible hybrids provide promising candidates for identifying transcripts involved in disease resistance. A microarray containing 62,876 UniGene transcripts selected from the CitEST database and prepared by NimbleGen Systems was used for analyzing global gene expression 48 hours after infection with *P. parasitica*.

**Results:**

Three pairs of data comparisons (*P. trifoliata*/*C. sunki*, Pool R/*C. sunki *and Pool R/Pool S) were performed. With a filter of false-discovery rate less than 0.05 and fold change greater than 3.0, 21 UniGene transcripts common to the three pairwise comparative were found to be up-regulated, and 3 UniGene transcripts were down-regulated. Among them, our results indicated that the selected transcripts were probably involved in the whole process of plant defense responses to pathogen attack, including transcriptional regulation, signaling, activation of defense genes participating in HR, single dominant genes (*R gene*) such as TIR-NBS-LRR and RPS4 and switch of defense-related metabolism pathway. Differentially expressed genes were validated by RT-qPCR in susceptible and resistant plants and between inoculated and uninoculated control plants

**Conclusions:**

Twenty four UniGene transcripts were identified as candidate genes for *Citrus *response to *P. parasitica*. UniGene transcripts were likely to be involved in disease resistance, such as genes potentially involved in secondary metabolite synthesis, intracellular osmotic adjustment, signal transduction pathways of cell death, oxidative burst and defense gene expression. Furthermore, our microarray data suggest another type of resistance in *Citrus*-*Phytophthora *interaction conferred by single dominant genes (*R gene*) since we encountered two previously reported *R genes *(TIR-NBS-LRR and RPS4) upregulated in the resistant genotypes relative to susceptible. We identified 7 transcripts with homology in other plants but yet unclear functional characterization which are an interesting pool for further analyses and 3 transcripts where no significant similarity was found. This is the first microarray study addressing an evaluation of transcriptional changes in response to *P. parasitica *in *Citrus*.

## Background

*Phytophthora nicotianae *Breda de Haan (*Phytophthora parasitica *Dastur) and *Phytophthora citrophthora *(Smith & Smith) have caused severe damage in *Citrus *nurseries and orchards worldwide. In Brazil, *P. parasitica *is the predominant species associated with the disease, found in more than 95% of groves and nurseries [1]. These pathogens infect the main scaffold branches of the tree, inducing the formation of cankers with gum exudation and the expansion of the lesions upwards affects secondary branches, while downward expansion affects the trunk and roots [2]. Infected trees usually lack vigor and may die prematurely [3]. *P. parasitica *is an oomycete, belonging to the kingdom Stremenopiles, which comprises a diverse group of organisms that has been consolidated as a result of analysis of mitochondrial and ribosomal DNA sequences [4]. These pathogens establish intimate relations with their hosts by forming haustoria during the infection, which are structures used for obtaining nutrients from the plant, redirecting host metabolism and suppressing host defence in biotrophy [5]. *P. parasitica *is considered a hemibiotroph, and therefore it first establishes itself in host tissues as a biotroph but then switches to a more necrotrophic type of growth, rapidly invading and killing host cells.

Selection and breeding for resistance to *Phytophthora *in citrus species is considered the most efficient approach to control the disease, since there are varying degrees of resistance within the genera *Citrus *and its relatives. In this context, *Poncirus trifoliata *is an important genotype because of its agronomic valuable characteristics including resistance to *Phytophthora*, Citrus Tristeza Virus (CTV) and citrus nematode (*Tylenchulus semipenetrans*) [6].

In response to pathogen attack, a series of plant defense responses leading to the hypersensitive reaction, cell wall modifications, production of reactive oxygen species (ROS), accumulation of phytoalexins, and synthesis of pathogenesis related (PR) proteins may be activated [7]. In general, the salicylic acid (SA)-dependent signaling leads to expression of PR proteins, the production of ROS, and localized cell death. This leads to a defense that is effective against biotrophs because it restricts pathogen growth via hypersensitive death of the infected cells. The same reaction allows growth of a necrotrophic pathogen. Against necrotrophic pathogens, jasmonic acid (JA) and ethylene (ET)-dependent defenses are successfully employed in plants activating a different set of PR proteins [8]. But there is evidence for extensive cross-talk between signaling pathways involving antagonistic and synergistic interactions [9]. In these aspects, it is interesting to investigate the plant response mechanisms in the presence of hemibiotrophic pathogens like *P. parasitica*, since they comprise both lifestyles during their development.

Biotechnology tools associated with conventional citrus breeding programs have facilitated the development of cultivars with desirable characteristics [10]. Microarray technology has been used to identify gene expression changes in several responses to stress, enabling comparison of transcript levels for thousands of genes simultaneously, such as response of sweet orange (*Citrus sinensis*) to '*Candidatus Liberibacter *asiaticus' infection [7] and [11]. In citrus, the first transcript profiling data was reported by Shimada et al., [12] who constructed a cDNA microarray to monitor expression of mRNA during fruit development. Subsequently, several citrus DNA microarray platforms were developed, such as The Spanish Citrus Genomic Consortium. Affymetrix developed and released a citrus GeneChip containing 960,444 total 25- mer oligos in an 11 micron format (http://www.affymetrix.com/analysis/index.affx), based on the NCBI citrus EST collection [6].

A new approach to study hybrid vigor at the molecular scale is to survey gene expression as a phenotype in F_1 _hybrids and their parental [13]. Genes in hybrids are inherited from the parents; thus, variation in regulation of the genes often leads to variation in the level of gene expression in hybrids, which in turn may alter the phenotype of these hybrids [14]. Due to the sensitivity of microarrays, plant-to-plant variation of gene expression could be reduced by bulk harvesting of resistant and susceptible hybrids. According to Wenger et al., [15] pooling segregants based on their phenotype allows the region of the genome responsible for the phenotype to be detected because DNA polymorphisms in regions unlinked to the responsible locus will segregate randomly and be ''evened'' out, while sequences or polymorphisms either directly responsible for the trait, or very closely linked to it, will be present in all positive segregants and absent in all negative segregants. According to Meng et al., [16] the majority of DNA microarrays in use today are created from single genomes that do not reflect the genetic diversity of a heterogeneous group. One alternative approach is to incorporate genetic information from several species within a single microarray slide. Mixed-DNA microarrays can be used to quickly assess the distribution of genetic diversity across multiple species.

In previous studies, four resistant and four susceptible F_1 _hybrids were selected from the population derived from a cross between *Citrus sunki *Hort. ex. Tan. and *P. trifoliata *(L.) Raf cv. Rubidoux, which are respectively, susceptible and resistant to *P. parasitica *[17,18]. We proposed that genes differentially expressed between resistant and susceptible parents and between their resistant and susceptible hybrids provide promising candidates for identifying transcripts involved in disease resistance. In the present study, we investigated gene expression in resistant and susceptible hybrids in comparison with their parents 48 hours after *P. parasitica *infection using a microarray chip containing 62,876 UniGene transcripts selected from *P. trifoliata*, *C. sinensis and C. reticulata *libraries. Five differentially expressed genes were validated by reverse transcription quantitative real-time PCR (RT-qPCR) in susceptible and resistant plants and used to detect differences in expression between inoculated and uninoculated control plants.

## Methods

### Plant material and inoculation

In a previous study, four resistant and four susceptible five-year-old hybrids (F_1 _population of *Citrus sunki *Hort. ex. Tan. × *Poncirus trifoliata *(L.) Raf cv. Rubidoux) were selected from 314 field-grown plants. The selected hybrids were the most resistant and susceptible to *P. parasitica *infection according to Boava [17] and Siviero *et al*. [18]. Three buds from each hybrid and parental line were collected and grafted onto 6-month-old Rangpur lime rootstocks. After six months, plants were inoculated by a mycelia disc; a mycelial block was placed onto the center of a cut made in the stem and covered with Parafilm. After 48 hours, leaves were harvested individually, separately flash-frozen in liquid nitrogen, and then stored at 80°C prior to RNA isolation. The selection of this harvesting time was based in previous studies on the time course of the type local and systemic defense followed by activation of many genes involved in the interaction Citrus-*Phytophthora*, detailed by Teixeira [19]. All inoculated plants were kept in a greenhouse at an average temperature of 25°C and 90% (± 0.5%) relative humidity until the final evaluation, measurement of the lesion size, at 40 days postinoculation. Experimental design was completely randomized including three biological replicates for each parent and three for each individual hybrid.

In addition, in another experiment with compatible plant-pathogen interactions, we used the *P. trifoliata *genotypes to detect differences in gene expression level between inoculated and uninoculated control plants. The plants for these experiments were grown and inoculated independently from microarray experiments. In this new experiment, we collected leaves of *P. trifoliata *for RNA isolations at 48 hours after *P. parasitica *inoculation and uninoculated control plants Experimental design was completely randomized including three biological replicates.

### Isolation of total RNA and sample labeling

Total RNA was isolated with RNeasy Plant Mini Kit (Qiagen) and treated with RNase-free DNase (Qiagen) according to the manufacturer's instructions. The concentration of total RNA was determined using a NanoDrop ND-1000 spectrophotometer (NanoDrop Technologies). RNA integrity was verified using a Bioanalyzer 1000 (Agilent). Following RNA isolation, the samples were pooled into resistant (Pool R) and susceptible hybrids (Pool S) to minimize variation between individual RNA samples. Three biological replicates of each parent and each resistant and susceptible pooled hybrids were used for hybridization with a cDNA microarray. RNA samples were sent to Roche NimbleGen Systems, where cDNA synthesis and Cy3 labeling were performed. Equal amounts of total RNA for each sample were converted to double-stranded cDNA using the SuperScript II cDNA Conversion Kit (Invitrogen). Because this method uses an oligo (dT) primer, RNA strands lacking poly(A) tails are likely to be underrepresented. Cy3 labeling, hybridization and data acquisition were performed at the NimbleGen facility following the manufacturer's procedures.

### Microarray data analysis

A total of 62,876 UniGene transcripts (31,583 of *C. sinensis*, 18,712 of *C. reticulata *and 12,581 of *P. trifoliata*) selected from the CitEST database, assembled from the ESTs submitted to NCBI (GenBank accession numbers EY649559 to EY842485) were used to construct oligonucleotide microarray chips by Roche NimbleGen Systems using a multi-step approach to select probes with optimal predicted hybridization characteristics. Three probes were selected per UniGene, comprising a probe set, and each probe set is represented on the final array by two replicates. All probes were designed as perfect match oligonucleotides. The experimental design and all microarray data have been deposited in the NCBI Gene Expression Omnibus (GEO, http://www.ncbi.nlm.nih.gov/geo accession number GSE20412). Arrays were hybridized and processed by Roche NimbleGen Systems as previously described [20]. For each resistant and susceptible hybrid pool and each parent sample, hybridization was performed on independent microarrays. Arrays were scanned by Roche NimbleGen using a GenePix 4000B microarray scanner (Molecular Devices, Sunnyvale, CA) and the data were extracted using NimbleScan software. For each probe set, an expression measure was calculated using the robust multiarray average (RMA) [21], consisting of three preprocessing steps: convolution background correction, quantile normalization [22], and a summarization based on a robust multiarray model fit using the median polish algorithm. Probe set data with these normalized expression values, provided by Roche NimbleGen Systems in RMA calls files, were imported to ArrayStar software 3.0 version (DNASTAR Inc., Madison, WI), where statistical analysis was performed.

Three pairs of data comparisons that might reveal an association with the resistance of citrus to *P. parasitica*, were performed: (i) *P. trifoliata *Rubidoux versus *C. sunki*; (ii) resistant hybrids (Pool R) versus *C. sunki*; and (iii) resistant hybrids (Pool R) versus susceptible hybrids (Pool S). For each comparison, a moderated t-test value was calculated, and p-values were adjusted for multiple comparisons by the false-discovery rate correction [23]. Afterwards, UniGene transcripts that were consistently differentially expressed (greater than 3.0-fold up or less than 0.33-fold down; p-value ≤ 0.05) between replicate samples were identified as involved in disease resistance. In order to eliminate UniGene transcripts selected as a consequence of genotype differences but not directly related to disease resistance, Venn diagrams were used to identify the intersection among these three sets of informative transcripts. These transcripts were then rechecked by BLASTX searches against the GenBank database, and further classified into categories according to the Munich Information Center for Protein Sequences classification system (MIPS; http://www.helmholtz-muenchen.de/mips).

### Real time-qPCR

Reverse transcription quantitative real-time PCR (RT-qPCR) was performed with five selected genes to validate the microarray experiments (Table 1). Our candidate gene analysis focused on upregulated UniGene transcripts in all comparison: *P. trifoliata *relative to *C. sunki*, in the resistant pool relative to *C. sunki*, and in the resistant pool relative to the susceptible pool. These candidate genes were judged to be biologically interesting on the basis of their predicted function retrieved from CitEST. These selected genes were also used to detect differences in expression between inoculated and uninoculated control plants.

**Table 1:** Oligonucleotide primers used for RT-qPCR analysis.

	Description	CitEST	Forward/Reverse	Amp
**Selected genes to validate the microarray**				
LEA	Lea 5	CAS-PT-303903	TCGGACTGGTATCATGGA/GTAGTACCCAGTGATGGGA	99
MIR	miraculin	CAS-CR-215276	AGCCCTGTAATGAAGAACC/TAGCAACGTTTCAGCTCC	100
TIR	TIR-NBS-LRR	CAS-CS-103511	CATGATGAGGACGTGGG/AAGTGATCCGACTCGAC	103
RPS4	Disease resistance RPS4	CAS-PT-300852	CCAAGATCTTGAATATCTTCCC/GCAAGTTGAGCTCAATTAGG	112
UNC	unclassified proteins	CAS-PT-310250	TCTCTTGTTCTTCATGCAGT/TATGCATCTTGCCTTCATTC	116
**Selected endogenous control genes**				
ETEF2	eukaryotic translation elongation factor 2	CAS-PT-306679	TTGAGGCTTCTGAATCGAG/CTTTCCAGATGAACCTCTCC	97
EGIDH	NADP-isocitrate dehydrogenase	CAS-CS-112964	CATTGAACATGCAGTTGAGG/ATTCTCATGACGTGTCGG	91
CYC	cyclophilin	CAS-PT-301486	AGAGTATGCAGAGGAATGG/GTCCTTAACAGAAGTCCGT	107
UBQ	ubiquitin	CAS-PT-300961	TTCGTCAGTTGACTAATCCT/GTTGCTGTGTTGACTGTG	95
TUB	tubulin	-	TTTGTAAGATCCCTCCGA/TCACCCTCCTGAACATTT	87

In order to find a reference gene to normalize the RT-qPCR results, the stability of five endogenous control genes in *Citrus *was analyzed to confirm their stability according to geNorm software [24] and to ensure the existence of gene expression variation due to the experimental conditions. Oligonucleotides primers were designed using *Primer Express *2.0 software (Applied Biosystems). From the RNA isolated as described above, cDNAs were synthesized from 3.0 μg of total RNA using Superscript III (200 U/μL) (Invitrogen) with an oligo (dT) primer (dT_12-18_, Invitrogen) according to the manufacturer's instructions. cDNA was treated with RNAse H (1 μl) for 20 min at 37°C to remove any contaminating RNA. RT-qPCR was performed using Power SYBR^® ^Green PCR Master Mix reagent (Applied Biosystems). The reaction consisted of 2.0 μL of cDNA and 120 nM of each gene-specific primer in a final volume of 15 μL. Amplification was carried out for three technical replicates for each sample, including negative controls. An ABI PRISM 7500 SDS (Applied Biosystems) was used for the following thermal cycles: 50°C for 2 min, 95°C for 10 min; 40 cycles of 95°C for 15 s, and 60°C for 1 min. Expression levels were assessed based on the number of amplification cycles needed to reach a common fixed threshold (cycle threshold - Ct) in the exponential phase of PCR. Ct data were analyzed using the GenEx version 4.3.6 software (http://www.multid.se/). For relative quantification, the 2^-ΔΔ^^*CT *^method between conditions in RT-qPCR was applied [25].

## Results and Discussion

### Response to infection by *P. parasitica*

**Figure 1:**
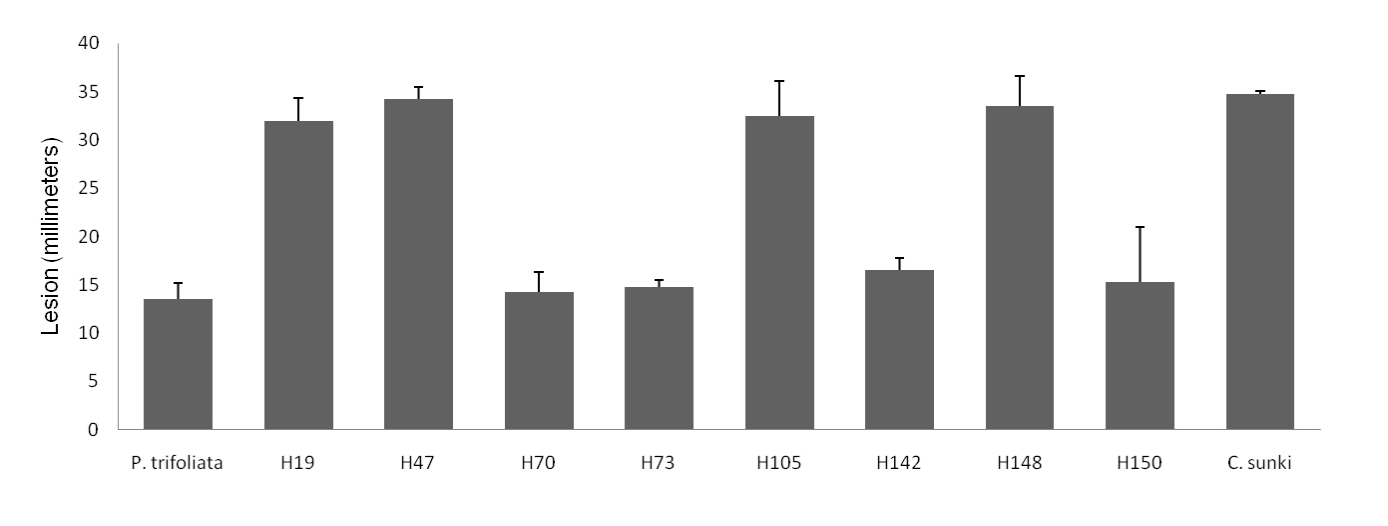
**Mean longitudinal length of the lesion (in mm) caused by *P. parasitica*, in resistant (H70, H73, H142, H150) and susceptible (H19, H47, H105, H148) F_1 _hybrids, and in their parents *Citrus sunki *and *Poncirus trifoliata *Rubidoux 48 hours after inoculation**. Vertical bars represent standard deviations of the means of three replicates.

Resistant and susceptible F_1 _hybrids of a population consisting of recombinants derived from a cross between *C. sunki *and *P. trifoliata *Rubidoux, respectively susceptible and resistant to *P. parasitica*, were evaluated in response to infection by *P. parasitica*. The F_1 _population was initially developed for mapping genetic loci for resistance against the citrus tristeza virus [26] and against *Phytophthora *gummosis [18]. This population was a good choice for mapping resistance to *Phytophthora *because the *Poncirus *genera possesses genes conferring many agriculturally important traits not found in *Citrus*, including genes responsible for resistance to *Phytophthora *[27]. In the present study, four selected resistant hybrids (H70, H73, H142, H150) and four selected susceptible hybrids (H19, H47, H105, H148) and their parents lines were inoculated by the disc method. The parents represented the extremes in lesion lengths, 13.5 mm for *P. trifoliata *and 33.75 mm for *C. sunki*, 40 days after *P. parasitica *inoculation. The F_1 _hybrids showed substantial differences in lesion lengths between resistant hybrids and susceptible hybrids (Figure 1). The four resistant hybrids and four susceptible ones showed respectively means of lesion size of 15.18 and 33.06 mm. These results were in accordance with previous studies carried out by [17,18]

### Microarray analysis

**Figure 2:**
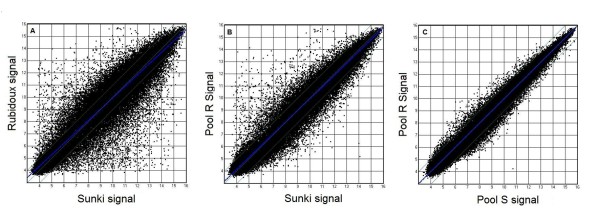
**Analysis of differential gene expression in *P. trifoliata *'Rubidoux', *C. sunki*, resistant (Pool R) and susceptible (Pool S) hybrids**. Signal correlation plots were used to examine comparisons between: (A) The average signal derived from the three biological replicates of resistant parent (Rubidoux) (Y-axis) and susceptible parent (Sunki) (X-axis) graphed on a logarithmic (base 2) scale. Note the prevalence of genes showing distinct expression patterns in the two genotypes; (B) A similar graph was made to compare expression in the resistant hybrids pool (Pool R) (Y-axis) and the susceptible parent (Sunki) (X-axis); (C) A similar graph of expression data of the resistant hybrids pool (Y-axis) plotted against the expression data of the susceptible hybrids pool (X-axis).

We proposed that genes differentially expressed between resistant and susceptible parents and between their resistant and susceptible hybrids provide promising candidates for identifying transcripts involved in citrus resistance against *Phytophthora. *Oligonucleotide microarray chips prepared by Roche NimbleGen Systems were used to profile the expression patterns. Expression was detected in tissues for many of the UniGene transcripts (a total of 62,876) present on the array selected from the CitEST database. The data were analyzed based on the RMA-processed expression values. Three possible comparisons (*P trifoliata*/*C. sunki*, Pool R/*C. sunki *and Pool R/Pool S) were analyzed individually using the log (base 2)-transformed normalized expression values as input data. Based on the criterion for differentially expressed, the transcripts were considered up- or down-regulated if the log2 ratio of resistant genotypes inoculated to susceptible genotypes results were greater (positive) than or less than (negative) 3.0-fold, respectively and *p*-value less than or equal to the level of significance α = 0.05. Thus, 6,735 UniGene transcripts (10.71%) were selected as differentially expressed in *P. trifoliata *relative to *C. sunki *(Figure 2A; Table 2), while 1,296 (2.06%) UniGene transcripts were selected as differentially expressed in resistant pool relative to *C. sunki *(Figure 2B; Table 2). When comparing the resistant with the susceptible hybrids pools, 564 (0,90%) transcripts were selected as differentially expressed (Figure 2C; Table 2).

**Table 2:** Comparisons between different genotypes inoculated with *P. parasitica *(*P. trifoliata*/*C. sunki*, Pool R/*C. sunki*, and Pool R/Pool S).

**Comparison**			
	
	**Total**	**Up**	**Down**
	
*P. trifoliata*/*C. sunki*	6,735	3,392	3,343
Pool R/*C. sunki*	1,296	870	426
Pool R/Pool S	564	205	359

The comparisons were analyzed individually. Genes greater (positive) than or less than (negative) 3.0-fold, respectively and p-value less than or equal to the level of significance α = 0.05 were considered differentially expressed.

**Figure 3:**
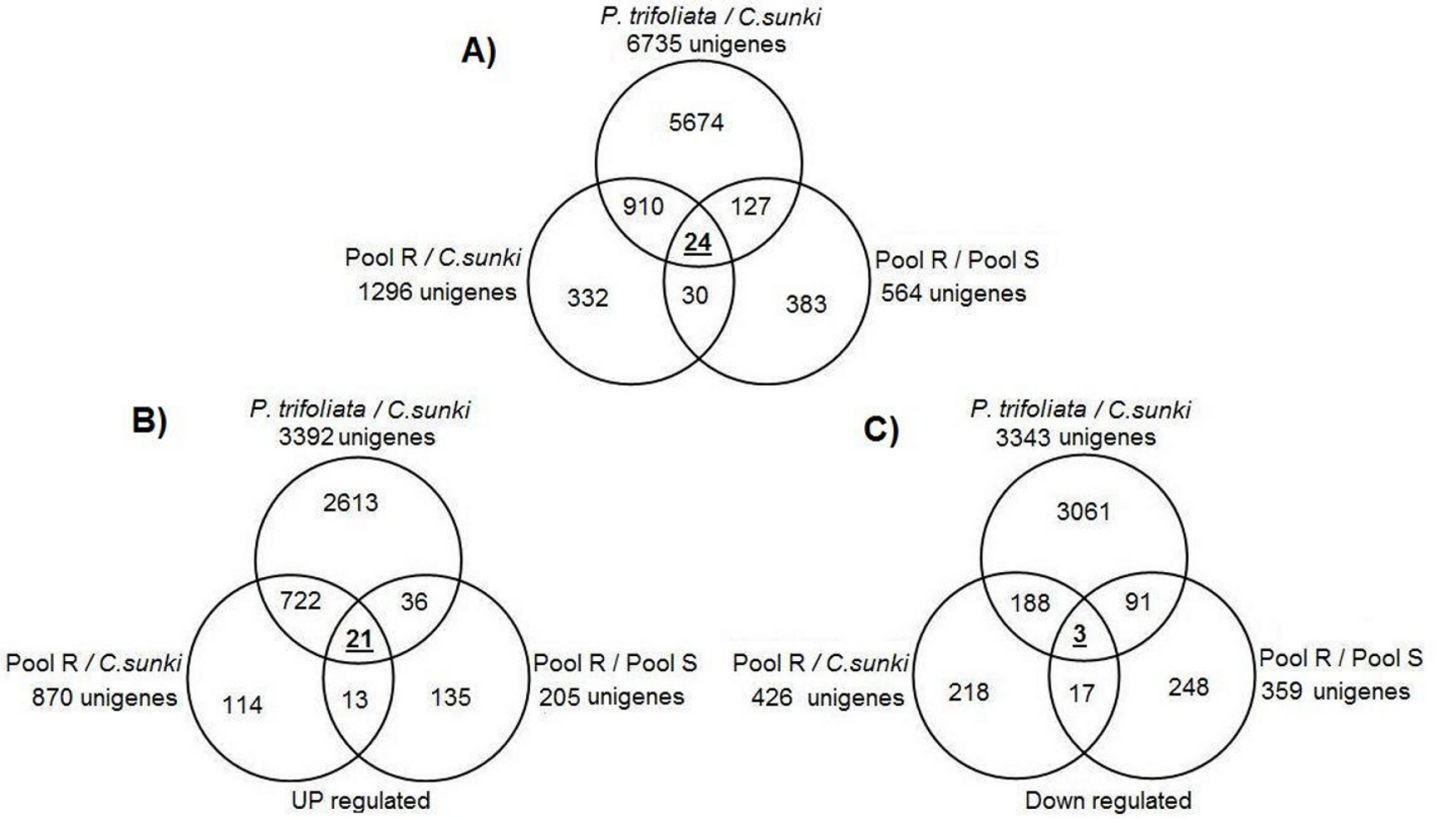
**Venn diagrams showing the differentially expressed UniGene transcripts in three different comparisons between resistant (Pool R) and susceptible (Pool S) F_1 _hybrids and their parents, *Citrus sunki *(Sunki) and *Poncirus trifoliata *Rubidoux (Rub), respectively susceptible and resistant to *P. parasitica*, 48 hours after inoculation**. Transcripts profile greater (positive) than or less than (negative) 3.0-fold, respectively and p-value less than or equal to the level of significance α = 0.05 were considered differentially expressed. A) differentially expressed UniGene; B) upregulated; C) downregulated

In addition, we observed differences in the expression levels in the resistant and susceptible parental genotypes and associated those with the differences in the expression levels in the resistant (pool R) and susceptible (pool S) hybrids. This association was accomplished by restricting the sets of differentially expressed UniGene transcripts to those identified as up- and downregulated in all three pairwise comparisons, Venn diagrams were used to identify the intersection among these three sets of informative transcripts, which resulted in the selection of 24 UniGene transcripts common to the three pairwise comparative analyses (Figure 3A, Table 3) of which 21 were up-regulated (Figure 3B, Table 3) and 3 were down-regulated (Figure 3C, Table 3) during *P. parasitica *infection, which were further classified into 7 categories according to the MIPS classification scheme (Table 3). The experimental design was in fact enriched with transcripts involved in pathogen response belonging to the categories disease/defense. We identified 11 UniGene transcripts that were likely to be involved in disease resistance; 7 transcripts with homology in other plants but yet unclear functional characterization which are an interesting pool for further analyses and 3 transcripts where no significant similarity was found.

**Table 3:** Differentially expressed UniGene transcripts in three different comparisons

ID CITEST	GENE_INFO	accession number	Categories (MIPS)	Rub × Sun		Pool R × Sunk		Pool R × Pool S	
				
Upregulated				Fold	P_Value	Fold	P_Value	Fold	P_Value
CAS-PT-300852	Disease resistance protein RPS4	BAB11393.1	cell rescue, defense and virulence	4,0	0,01	5,0	0,00	3,0	0,00
CAS-CS-103511	TIR-NBS-LRR resistance protein	XP_002325496.1	cell rescue, defense and virulence	4,0	0,02	4,2	0,02	4,0	0,01
CAS-PT-303903	Lea5 protein	Q39644.1	cell rescue, defense and virulence	30,9	0,00	22,0	0,00	4,1	0,01
CAS-PT-301521	FNR2 (ferredoxin-NADP(+)	NP_001077566.1	cell type localisation	13,5	0,02	13,2	0,03	3,3	0,02
CAS-CS-115011	Leucoanthocyanidin dioxygenase	XP_002528475.1	metabolism	5,3	0,03	6,4	0,04	3,2	0,01
CAS-CS-103575	Miraculin-like protein 2	ACL78790.1	protein activity regulation	43,1	0,01	25,1	0,02	10,1	0,01
CAS-CR-209520	AL07-2p	ACJ03067.1	protein fate (folding, mod., destination)	87,5	0,00	106,4	0,02	3,7	0,00
CAS-CS-119563	Serine-threonine protein kinase	XP_002514954.1	protein fate (folding, mod., destination)	86,1	0,01	162,3	0,02	7,9	0,00
CAS-CS-122102	Serine-threonine protein kinase	XP_002512394.1	protein fate (folding, mod., destination)	25,5	0,01	50,8	0,04	4,6	0,02
CAS-CS-128948	Transducin family protein	ABS32230.1	protein with binding function	6,8	0,04	4,0	0,02	3,1	0,05
CAS-CS-118778	Synaptobrevin-related family	XP_002304221.1	subcellular localisation	5,6	0,02	5,6	0,04	4,4	0,05
CAS-PT-303418	Putative DNA binding protein	ABO93454.1	subcellular localisation	10,7	0,00	9,9	0,02	3,1	0,03
CAS-PT-305376	Gag-pol polyprotein	AAR13298.1	transposable elements	17,2	0,04	3,7	0,02	3,3	0,02
CAS-PT-309741	Hypothetical protein	XP_002609958.1	unclassified proteins	18,7	0,01	12,0	0,05	3,3	0,01
CAS-PT-309581	Hypothetical protein	XP_001018346.1	unclassified proteins	24,9	0,00	27,1	0,03	7,0	0,01
CAS-CS-114114	Hypothetical protein	XP_002280912.1	unclassified proteins	19,6	0,02	29,4	0,04	3,1	0,01
CAS-PT-310125	Hypothetical protein	XP_002275595.1	unclassified proteins	13,1	0,01	28,4	0,01	5,4	0,01
CAS-CS-105219	Hypothetical protein	XP_002590734.1	unclassified proteins	6,6	0,03	6,2	0,02	3,1	0,02
CAS-PT-305253	No significant similarity found		unclassified proteins	25,5	0,01	6,2	0,05	3,2	0,01
CAS-CS-127334	No significant similarity found		unclassified proteins	10,2	0,00	6,2	0,04	3,6	0,00
CAS-PT-301031	No significant similarity found		unclassified proteins	70,6	0,01	37,6	0,01	3,4	0,01
**Downregulated**									
CAS-CS-101776	superoxide dismutase	CAA03881.1	function or cofactor requirement	4,0	0,02	4,2	0,02	4,0	0,01
CAS-CS-114557	predicted protein	XP_002324594.1	unclassified proteins	30,9	0,00	22,0	0,00	4,1	0,01
CAS-CR-203746	predicted protein	XP_002331132.1	unclassified proteins	13,5	0,02	13,2	0,03	3,3	0,02

*P. trifoliata *Rubidoux versus *C. sunki*; resistant hybrids (Pool R) versus *C. sunki*; and resistant hybrids (Pool R) versus susceptible hybrids (Pool S), 48 hours after *P. Parasitica *inoculation.

The 24 UniGene transcripts common to the three pairwise comparative analyses were distributed among the 3 different libraries used for built of the our microarray chip from the CitEST database, 12 UniGene transcripts were identified in the UniGene set of *C. sinensis*, 10 in *P. trifoliata*, and 2 in *C. reticulate*. According to Wan *et al. *[28], the use of mixed-DNA microarrays is advantageous because much more information is incorporated into the analysis. In the present work, there were 18,712 UniGene transcripts from *P. trifoliata *libraries of the CitEST database and we decided to incorporate UniGene sequences from *C. sinensis *and *C. reticulata *to be represented in the array and consequently enrich the analyses. The Spanish Citrus Genomic Consortium also developed a mixed-DNA microarrays composed of 24,000- element cDNA array containing 20,000 unigenes, based on nearly 90,000 high-quality sequences generated from 52 different cDNA libraries [6].

### Real time-qPCR validation

**Figure 4:**
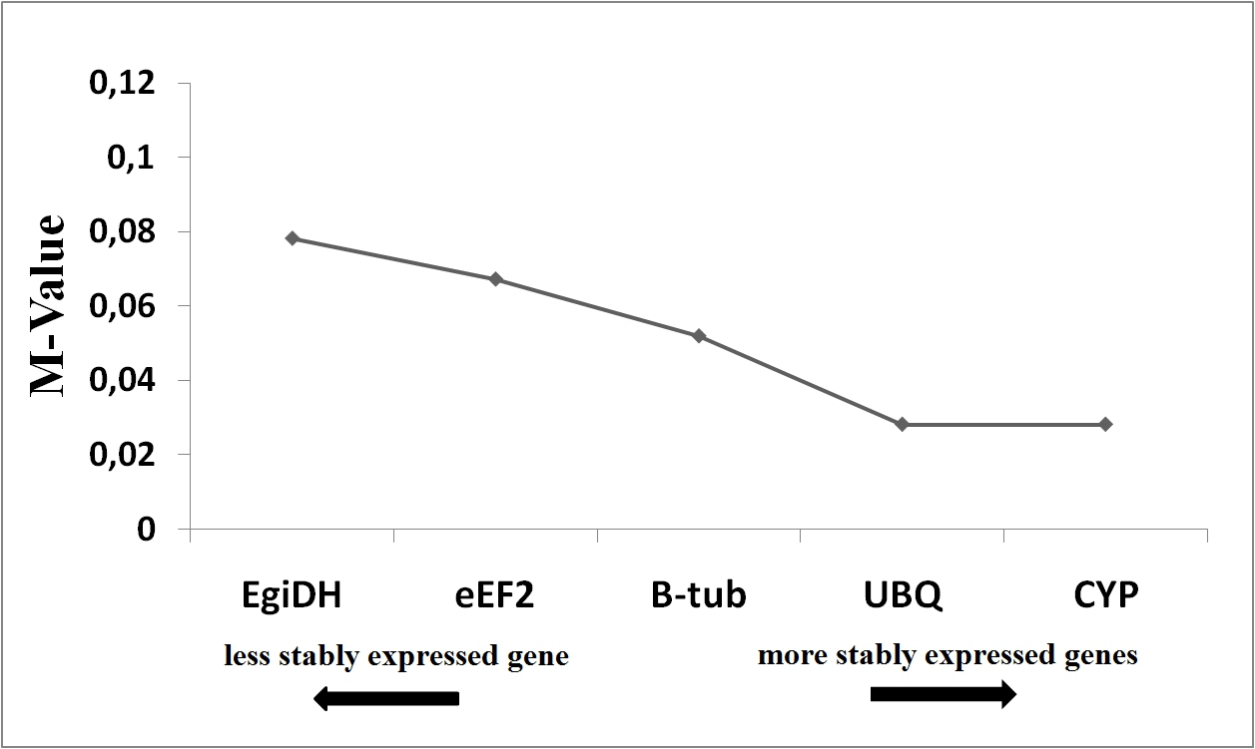
**Expression stability mean values (M-values) of 5 endogenous control genes, in tissue samples from citrus genotypes 48 hours after *P. parasitica *inoculation after analysis using geNorm software**.

RT-qPCR is currently the most sensitive method to compare gene expression at both low and high levels. To avoid distortions, RT-qPCR requires a reference gene (or a few genes) with the most stable expression over the experiment, i.e., a gene presenting expression levels that is little influenced by the experimental or environmental conditions, to correct for sample-to-sample variation in RT-qPCR efficiency and errors in sample quantification. Several reference genes tested presented varied expression due to the experimental conditions in different genotypes exposed to biotic and abiotic stimuli [29]. To support the choice of the best reference genes, i.e., the most stable genes following pathogen inoculation in the different genotypes, the GeNorm algorithm [24], implemented in GenEx version 4.3.6. software (http://www.multid.se/) was used. In the present work, among the five reference genes tested (Table 1), ubiquitin (*UBQ*) and cyclophilin (*CYP*) had the lowest expression stability mean values (M-value = 0.0257), i.e., these genes had more stable expression than other evaluated genes (Figure 4), and the data were normalized by the normalization factor calculated using these most stable reference genes.

**Figure 5:**
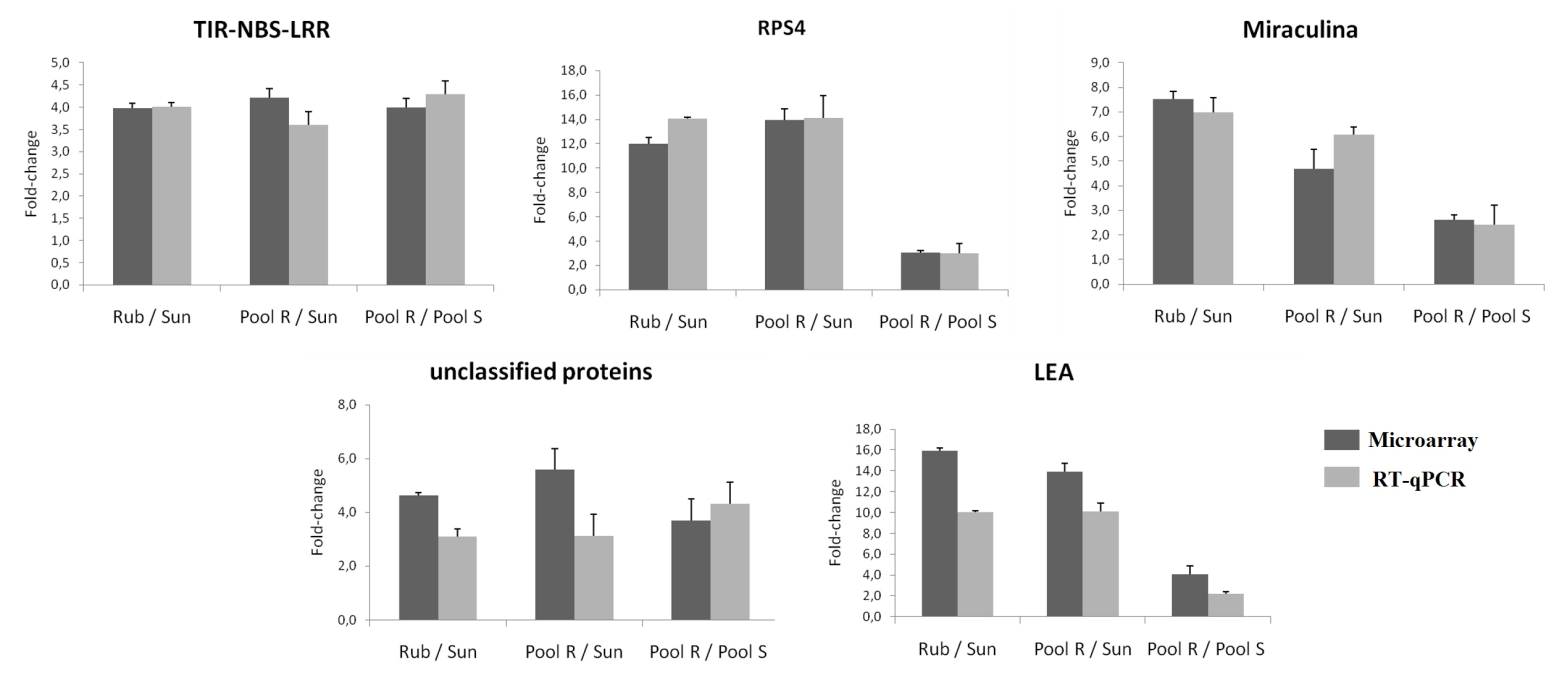
**Validation of microarray data by quantitative real-time**. RT-qPCR fold-changes are shown for five genes upregulated in all microarray pairwise comparisons (Rub/Sun, Pool R/Sun, and Pool R/Pool S) 48 hours after *P. parasitica *inoculation, and compared with fold-changes obtained by microarray analysis. RT-qPCR data were normalized to the two most stable endogenous control genes (*UBQ *and *CYP*).

Five UniGene transcripts that were differentially expressed in the microarray analysis were selected for RT-qPCR. Our candidate gene analysis focused on upregulated genes in the resistant genotypes that were judged to be biologically interesting on the basis of their predicted function retrieved from CitEST. We selected genes that are likely involved in disease resistance, such as one gene potentially involved in secondary metabolite synthesis and protein activity regulation (miraculina) and genes potentially involved in cell rescue, defense and virulence (TIR-NBS-LRR and RPS4) and one gene homolog in other plant but yet unclear functional characterization which are an interesting for further analyses. Comparison of the results from RT-qPCR with those from microarray analyses revealed roughly similar patterns or tendencies of expression in the three comparative pairwise analyses (Figure 5). Notably, using high stringency in the analysis of the microarray, data showed only twenty one genes were upregulated in common among the three comparative pairwise analyses. The validation with RT-qPCR showed that all the selected genes were in fact upregulated to similar levels in all comparison (≥ 3.0-fold, p-value ≤ 0.05). All five transcripts were upregulated in *P. trifoliata *relative to *C. sunki*, in the resistant pool relative to *C. sunki*, and in the resistant pool relative to the susceptible pool. These results revealed similar patterns of expression with those from microarray analyses.

**Figure 6:**
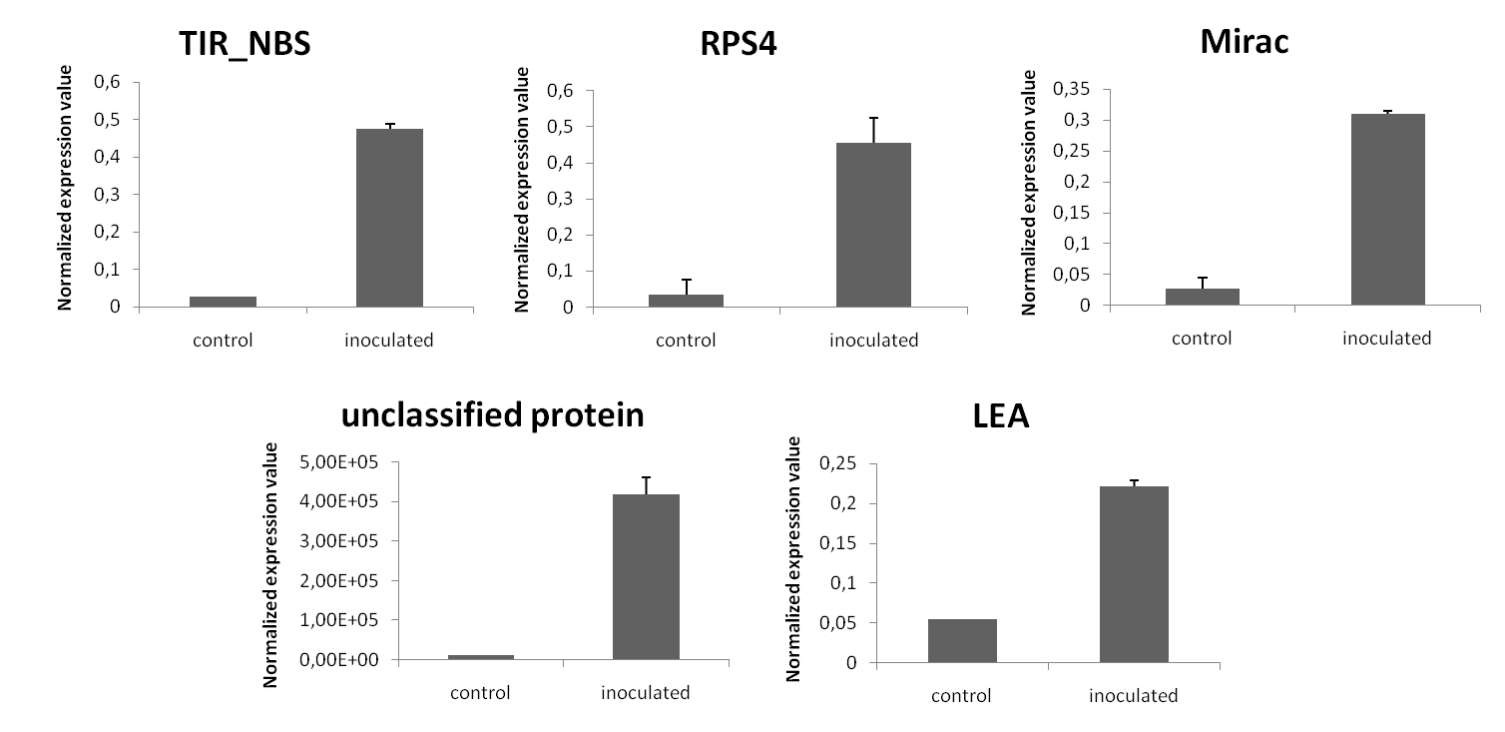
**Validation of microarray data by RT-qPCR using *P. trifoliata *parents uninoculated control and inoculated with *P. parasitica *after 48 hours**. RT-qPCR data were normalized to the two most stable candidate endogenous control genes (*UBQ *and *CYP*).

In addition, besides of estimating and confirm the fold change at each comparative pairwise analyses on microarray, we include in our RT-qPCR analysis, five selected genes in inoculated and uninoculated plants of *P. trifoliata*. (Figure 6). Thus, this confirmation offers an opportunity to determine transcription patterns of the systemic response of *Citrus *to infection with the pathogen *P. parasitica *providing information on different defense and metabolic pathways. With this analysis the differences in gene expression probably are involved in the response to infection to *P. parasitica *and maybe some of them can be related to the resistance mechanism and the changes found are not only due to inherent constitutive gene expression differences among the genotypes"

### Functional analyses of differentially-expressed genes

Upon recognition of *P. parasitica *by its host *Citrus*, a series of signaling pathways are switched on, which lead to the metabolic reprogramming of the host plant. *Phytophthora *species are usually biotrophic or hemibiotrophic pathogens that keep host cells alive (at least initially) to enable nutrient uptake from the plant cells [30]. A more extensive cell death response could cause opposite effects on the outcome of biotrophic versus necrotrophic plant-pathogen interactions. Hemibiotrophic pathogens adopt a two-step infection style. According to Kanneganti *et al. *[31], during the phase of infection that follows penetration of host tissue, they require living cells, much like biotrophic pathogens. In contrast, in a later phase of the disease, they cause extensive necrosis of host tissue, resulting in profuse colonization and sporulation. This infection cycle suggests that host cell death may impact the disease differently depending on its timing.

The time-point of infection was selected for the isolation of defense-related transcripts because, in compatible interactions, transcripts involved in pathogen response were observed at 48 hours (e.g., [19]) and this is reflected by the low number of transcripts of secondary metabolism isolated in this study. According to Grenville-Briggs and van West [32]*Phytophthora *species are hemibiotrophs being biotrophic for the initial stage of up to 36 h after inoculation. Molecular studies carried out with hemibiotrophic pathogens infecting plants helped the identification of several putative genes that are expressed at the stage biotrophy and are involved in membrane or cell wall biosynthesis, amino acid metabolism, osmoregulation, phosphorylation, protein secretion and energy consumption [33].

In our study, genes potentially involved in secondary metabolite synthesis were identified, including leucoanthocyanidin dioxygenase, which is the key enzyme leading to the synthesis of anthocyanins. Anthocyanins are known to be potent agents acting against oxidative stress [34]. The exact role of anthocyanins in defense is not clear but it could be that these compounds neutralize damaging reactive oxygen species (ROS) [35]. A late embryogenesis-abundant LEA5 protein was also found upregulated in the resistant genotypes. LEA proteins have been considered to play roles in maintaining membrane structures, binding of water, and acting as molecular chaperones [36]. According to Galau [37], these genes are believed to be induced in response to the expansion and maintenance of giant cells, and may play a role in intracellular osmotic adjustment. These results demonstrate that a transcriptional reprogramming has occurred within the first 48 hours after inoculation, which might correspond to the *P. parasitica *biotrophic phase.

Cell death triggered by ROS at the interface of necrotrophic fungus-plant interactions has been shown to be required for disease susceptibility [38]. Therefore, ROS in conjunction with *P. parasitica*-induced ET, may play a role in development of visible necrotrophic symptoms, observed 40 days after inoculation. Several results suggest that *P. parasitica *may produce a toxin-like virulence factor similar to necrosis and ET-inducing peptides characterized in other fungal pathogens such as *Phytophthora *spp. [39], *Botrytis *spp. [40] and *Pythium aphanidermatum*, during compatible-necrotrophic interactions. According to Attard et al. [41] all *Phytophthora *species abundantly secrete 10 kDa proteins, which form a superfamily called elicitins. The EST sequencing project for *P. parasitica *led to the identification of 10 different elicitin classes [42,43]. Elicitins have been found to be up regulated lately when the pathogen entered the necrotrophic growth stage [44].

Elicitins can induce a hypersensitive reaction (HR) and further defense mechanisms in the host [45]. In our study, we found two Unigenes transcripts encoding serine-threonine protein kinase upregulated in the resistant genotypes. According to Sasabe et al. [46] protein phosphorylation is an indispensable process for signal transduction pathways of cell death, oxidative burst and defense gene expression. One or more proteins whose activity is controlled by Ser/Thr protein kinases and phosphatases might be activated by elicitin-treatment, or kinase proteins which are sensitive to phosphatases might play an important role in cell death, oxidative burst and defense gene expression at the upstream of common signal transduction pathway and/or by independent pathways.

Sasabe et al. [46] report that *Phytophthora citricola *elicitin molecule induces apoptotic cell death in tobacco and they isolated several EST involved in plant oxidative or respiratory burst like superoxide dismutases and NADPH oxidase. Here, we identified one superoxide dismutase downregulated in the resistant genotypes, which were consequently upregulated in susceptible plants relative to resistant. Several enzymes, such as superoxide dismutase are responsible for the removal of production of signaling compounds such as reactive oxygen intermediates (ROIs) [47]. According to Mitler et al., [48] cell death of plant tissues resulted from attack by pathogens does not only occur in HR, resistant responses, but also in susceptible reactions such as necrotic symptom, for example which is caused by toxin. The late induction of elicitins observed in hemibiotrophic pathogen interaction [44] is therefore probably a component of *Phytophthora*'s pathogenic strategy under positive selection. This explains the late but extended formation of necrosis observed in this study in susceptible genotypes.

Our microarray data, which identified different defense and metabolic pathways, is congruent with the results from previous studies by Siviero et al [18]. These authors, studying of the mode of inheritance associated to the resistance against *Phytophthora *gummosis in *Poncirus trifoliata*, identified three quantitative trait loci associated to the resistance against *Phytophthora *gummosis in citrus, indicating the quantitative pattern of the disease. Because this resistance is controlled by multiple genes, the pathogen has to undergo multiple mutations to adapt to partial resistance which is more difficult than a single locus mutation. In this case, plants may exhibit resistance to most isolates to a certain degree by reducing pathogen reproduction, infection efficiency and colonization. This resistance is more durable than that mediated by resistance genes (R gene), but is difficult to move into cultivated varieties by crossing and phenotypic selection.

However, our microarray data suggest another type of resistance in *Citrus*-*Phytophthora *interaction, which resistance may be inherited qualitatively conferred by single dominant genes (*R gene*). We encountered two previously reported *R genes *(TIR-NBS-LRR and RPS4) upregulated in the resistant genotypes relative to susceptible. The largest class of R genes is characterized by the presence of nucleotide-binding site (NBS) sequence followed by leucine-rich repeats (LRRs) [49]. NBS-LRR class R genes, such as *RPS4 *in *Arabidopsis *can also be characterized by the presence of a leading sequence homologous to the TIR (Toll-Interleukin-1 receptor) that is responsible for cytoplasmic signaling in animals [49]. Previous data suggest that the TIR domain of RPS4 is important for induction of cell death when RPS4 is transiently expressed in tobacco leaves [50]. NBS-LRR-mediated resistance has been identified against numerous types of biotrophic or hemibiotrophic pathogens, including fungi, oomycetes, viruses and bacteria, and these types of resistance genes have been identified across a wide range of plants [51].

Qualitative resistance is mediated by R genes that lead to a race-specific hypersensitive response. These R genes only provide short-lived resistance in the field as new virulent races of the pathogen rapidly overcome the resistance encoded by single race-specific resistance genes [52]. In contrast, quantitative resistance is controlled by many interacting genes that do not prevent infection, but slow down the development of the pathogen at individual infection sites on the plant, and hence, lasts longer [53].

Although the presence of R gene does not contradict the statement that the *Poncirus *genera used in our experiments possess quantitative pattern of resistance to *P. parasitica *, these genes showed strong induction with an alteration of more than 4-fold in resistant genotypes relative to susceptible (Table 3). This finding further support the previous point of view that the HR was one of the main defense responses. R genes are also thought to encode specific receptors that recognize elicitors and initiate signal transduction cascades resulting in the HR [54]. Although a major feature of the HR is a rapid and local cell death, many defense-related genes that are not involved in cell death are also activated and may play a more important role in resistance to pathogen. Therefore, it is further inferred that the HR in resistant genotypes may result from a broad-spectrum recognition of the pathogen by the product of unknown R gene(s) or R gene analogues, or that a same or similar defense system, especially the downstream signaling components such as kinases and other defense genes, may exist specific resistances.

## Conclusion

This is the first global gene expression study in Citrus × *Phytophthora *gummosis interaction. We identified 24 *Phytophthora *gummosis responsive transcripts. The functional classification of these genes and their expression profile at 48 h after inoculation provide useful information on resistance of citrus to *Phytophthora*. The selected transcripts were validated by RT-qPCR in susceptible and resistant plants and between inoculated and uninoculated control plants. Our results indicated that the selected transcripts were really upregulated in the resistant genotypes and probably involved in the whole process of plant defense responses to pathogen attack, including transcriptional regulation, signaling, activation of defense genes participating in HR, such as TIR-NBS-LRR and RPS4, switch of defense-related metabolism pathway.

As we have a linkage map for *P. trifoliata *and three QTL for *Phytophthora *gummosis resistance localized on this map, the integration of genomics and genetic mapping using genetic genomics approaches will provide new insights into resistance to this disease and help with the development of improved disease management strategies. The genes that we have identified as upregulated across the resistant genotypes will be valuable for ongoing work in eQTL mapping.

## Authors' contributions

MCY and MAM planned and supervised the study. LPB, MCY, contributed to the design and execution of the experiments detailed. LPB and MCY carried out plant growth and inoculation. LPB, VSM and KK participated in extraction of plant RNA. LPB and MRA performed microarray analysis. LPB, VSM and KK contributed to the primer design and RT-qPCR validation. KTL performed annotations of the UniGene transcripts. LPB and MCY drafted the manuscript. MCY, MAT and MAM provided intellectual input. MAM, MRA, VSM, KK and MAT revised the manuscript. All authors read and approved the final manuscript.
